# High Blood Pressure and Long-Term Exposure to Indoor Noise and Air Pollution from Road Traffic

**DOI:** 10.1289/ehp.1307156

**Published:** 2014-07-08

**Authors:** Maria Foraster, Nino Künzli, Inmaculada Aguilera, Marcela Rivera, David Agis, Joan Vila, Laura Bouso, Alexandre Deltell, Jaume Marrugat, Rafel Ramos, Jordi Sunyer, Roberto Elosua, Xavier Basagaña

**Affiliations:** 1 Centre for Research in Environmental Epidemiology (CREAL), Barcelona, Spain; 2 CIBER Epidemiología y Salud Pública (CIBERESP), Barcelona, Spain; 3 Departament de Ciències Experimentals i de la Salut (UPF), Universitat Pompeu Fabra, Barcelona, Spain; 4 Swiss Tropical and Public Health Institute, Basel, Switzerland; 5 University of Basel, Basel, Switzerland; 6 University of Montreal Hospital Research Center (CRCHUM), Montréal, Quebec, Canada; 7 IMIM (Hospital del Mar Medical Research Institute), Barcelona, Spain; 8 GREFEMA (Grup de Recerca en Enginyeria de Fluids, Energia i Medi Ambient), Girona, Spain; 9 University of Girona (UdG), Girona, Spain; 10 Jordi Gol Institute for Primary Care Research (IDIAP-Jordi Gol), Girona Institute for Biomedical Research (IDIBGI), Catalan Institute of Health, Girona, Catalunya, Spain; 11 Department of Medical Sciences, School of Medicine, University of Girona, Girona, Spain

## Abstract

Background: Traffic noise has been associated with prevalence of hypertension, but reports are inconsistent for blood pressure (BP). To ascertain noise effects and to disentangle them from those suspected to be from traffic-related air pollution, it may be essential to estimate people’s noise exposure indoors in bedrooms.

Objectives: We analyzed associations between long-term exposure to indoor traffic noise in bedrooms and prevalent hypertension and systolic (SBP) and diastolic (DBP) BP, considering long-term exposure to outdoor nitrogen dioxide (NO_2_).

Methods: We evaluated 1,926 cohort participants at baseline (years 2003–2006; Girona, Spain). Outdoor annual average levels of nighttime traffic noise (*L*_night_) and NO_2_ were estimated at postal addresses with a detailed traffic noise model and a land-use regression model, respectively. Individual indoor traffic *L*_night_ levels were derived from outdoor *L*_night_ with application of insulations provided by reported noise-reducing factors. We assessed associations for hypertension and BP with multi-exposure logistic and linear regression models, respectively.

Results: Median levels were 27.1 dB(A) (indoor *L*_night_), 56.7 dB(A) (outdoor *L*_night_), and 26.8 μg/m^3^ (NO_2_). Spearman correlations between outdoor and indoor *L*_night_ with NO_2_ were 0.75 and 0.23, respectively. Indoor *L*_night_ was associated both with hypertension (OR = 1.06; 95% CI: 0.99, 1.13) and SBP (β = 0.72; 95% CI: 0.29, 1.15) per 5 dB(A); and NO_2_ was associated with hypertension (OR = 1.16; 95% CI: 0.99, 1.36), SBP (β = 1.23; 95% CI: 0.21, 2.25), and DBP (β⊇= 0.56; 95% CI: –0.03, 1.14) per 10 μg/m^3^. In the outdoor noise model, *L*_night_ was associated only with hypertension and NO_2_ with BP only. The indoor noise–SBP association was stronger and statistically significant with a threshold at 30 dB(A).

Conclusion: Long-term exposure to indoor traffic noise was associated with prevalent hypertension and SBP, independently of NO_2_. Associations were less consistent for outdoor traffic *L*_night_ and likely affected by collinearity.

Citation: Foraster M, Künzli N, Aguilera I, Rivera M, Agis D, Vila J, Bouso L, Deltell A, Marrugat J, Ramos R, Sunyer J, Elosua R, Basagaña X. 2014. High blood pressure and long-term exposure to indoor noise and air pollution from road traffic. Environ Health Perspect 122:1193–1200; http://dx.doi.org/10.1289/ehp.1307156

## Introduction

Long-term exposure to outdoor traffic noise has been associated with cardiovascular disease (CVD) (Babisch 2006). The biological pathway involves noise–stress reactions related to hormonal and cardiovascular responses that, under long-term exposure, may contribute to hypertension and CVD—particularly during susceptible periods such as sleep at night (Babisch 2011).

Hypertension is the leading risk factor for morbidity and mortality worldwide (Lim et al. 2012). A recent comprehensive meta-analysis reported an increase in prevalence of hypertension per 5-dB(A) increase in daytime traffic noise levels (*L*_Aeq,16h_) [range, 45–75 dB(A)] [odds ratio (OR) = 1.03; 95% confidence interval (CI): 1.01, 1.06] (van Kempen and Babisch 2012). However, studies on the association between long-term exposure to noise and the continuous trait of blood pressure (BP) are heterogeneous (Babisch 2006).

Traffic is also the primary source of local air pollution, and recent cross-sectional studies indicate associations between long-term exposure to markers of traffic-related pollution and high BP (Chuang et al. 2011; Dong et al. 2013; Foraster et al. 2014; Fuks et al. 2011; Schwartz et al. 2012). However, the evidence is still limited, particularly for hypertension (Coogan et al. 2012; Fuks et al. 2011; Sørensen et al. 2012).

A major unresolved concern is whether long-term effects of traffic-related air pollution and noise could be mutually confounded (Allen et al. 2009; Foraster et al. 2011). As emphasized in the literature (Babisch 2011), current studies rely on outdoor traffic noise estimates at the most exposed façade, whereas the true exposure may well differ depending on room orientation, noise shielding, and coping behaviors (Babisch et al. 2012). Understanding traffic noise exposure indoors, during sleep, could be essential to ascertaining the cardiovascular effects of noise and disentangling them from those of traffic-related air pollution.

We aimed to evaluate the association of long-term exposure to individually assigned estimates of indoor traffic noise levels in bedrooms at night (*L*_night_), a susceptible period to noise exposure, with BP and hypertension. To derive indoor levels, we combined outdoor traffic noise levels with information about the bedroom’s orientation and measures against noise. We also evaluated the confounding effect of traffic-related air pollution. The study was conducted within the well-defined population-based cohorts of the REGICOR (Registre Gironí del Cor; Girona Heart Registry) study in Girona, a dense Mediterranean city of nearly 100,000 inhabitants in northeast Spain.

## Methods

*Study sample*. The initial sample consisted of 2,067 participants, 36–82 years of age, who were evaluated at baseline (2003–2006) within a population-based cohort of the REGICOR study (Grau et al. 2007), and who had answered a questionnaire on nighttime noise exposure at the bedroom at follow-up (2009–2011). Briefly, the baseline sample was a random selection of noninstitutionalized inhabitants of Girona who were called in a randomized order for the follow-up visit. Because the noise questionnaire referred to the residence at follow-up, we selected nonmovers from baseline to follow-up (93.3% of the follow-up sample) to ensure that responses referred to the same baseline residences.

The study was approved by Parc de Salut Mar ethics committee, and participants signed written informed consent.

*Outcomes and health assessment*. Participants were examined from 0800 to 1100 hours at the primary care center and after fasting for 10 hr but being allowed regular medication. Trained nurses measured BP and heart rate following the Joint National Committee VII recommendations (Chobanian et al. 2003), in sitting position, and with a calibrated automatic device (OMRON 711; Omron Healthcare, Lake Forest, IL, USA). Two measurements were done after at least 10 and 3 min of rest, respectively. If measurements differed by ≥ 5 mmHg, a third one was taken. To minimize the “white coat” effect, we used the last measurement. The nurses also measured weight and height and drew blood. The samples were coded, shipped to a central laboratory, and frozen at –80°C until the assay. Serum glucose, total cholesterol, and triglycerides were determined by enzymatic methods (Roche Diagnostics, Basel, Switzerland) in a Cobas Mira Plus autoanalyzer (Roche Diagnostics). Whenever triglycerides were < 300 mg/dL, LDL (low-density lipoprotein) cholesterol was calculated by the Friedewald equation. Quality control was performed with the External Quality Assessment–WHO Lipid Program [World Health Organization (WHO), Prague, Czech Republic] and Monitrol–Quality Control Program (Baxter Diagnostics, Dudingen, Switzerland).

We defined hypertension as having systolic (SBP) or diastolic (DBP) BP levels ≥ 140/90 mmHg, respectively (Chobanian et al. 2003), or reporting antihypertensive treatment with a positive response to the question “Do you take or have you taken any doctor prescribed medication to reduce blood pressure in the last two weeks?” For BP analyses, we defined a variable accounting for any “BP-lowering medication,” which included the self-reported antihypertensive treatment defined above or the use of “antihypertensives” or “beta-blockers” as coded by a physician from the medication list provided by participants, namely diuretics, ACE (angiotensin-converting enzyme) inhibitors, alpha or beta-blockers, angiotensin receptor II blockers, and calcium channel blockers. This variable was coded by a physician from the medication list provided by participants.

*Exposure assessment*. We derived individual long-term average levels of nighttime traffic noise (*L*_night_, 2300 to 0700 hours) expressed in A-weighted decibels [dB(A)] at the geocoded residential addresses (hereafter called outdoor traffic *L*_night_). Geocodes were separated 2 m from the postal address’s façade and located at the floor’s height of each dwelling. We derived the estimates with a detailed and validated city-specific traffic noise model (year 2005), described elsewhere (Foraster et al. 2011). This model complies with the European Noise Directive 2002/49/EC (END) (European Parliament and Council of the European Union 2002) and uses the interim European method NMPB routes-96 [CERTU (Centre d’Études sur les Réseaux, les Transports, l’Urbanisme et les Constructions Publiques) et al. 1997]. Estimates were computed at each receptor point by numerical calculations using CadnaA software (DataKustik, Greifenberg, Germany). The main input variables were speed limit, street slopes, type of asphalt, urban topography, and traffic density, also for small streets based on the Good Practice Guidelines for noise mapping (European Commission Working Group Assessment of Exposure to Noise 2003). Because railway noise may also be associated with BP (Dratva et al. 2012), and a single railway crosses dense traffic areas from North to South, we also derived individual residential railway noise estimates (*L*_night_) from an END-based model according to the International Organization for Standardization (ISO; Geneva, Switzerland) standard 9613. The propagation model was built on source identification of railway noise with daytime and nighttime measurements of the noise frequencies (1/3-octave bands) and equivalent levels [in dB(A)] of freight and normal trains (a total of 72 measurements). Measurements were taken with an SC-30 sound level meter and a CB-5 calibrator (CESVA, Barcelona, Spain). Our study sample was not exposed to aircraft noise.

In a face-to-face interview we collected information on noise sensitivity (Weinstein 1978)—a 10-item score based on a nonverbal 6-point scale—and traffic noise annoyance (Fields et al. 2001)—nonverbal 11-point scales—in the bedroom during sleeping hours, as previously done (Babisch et al. 2012). We also evaluated *a*) type of glazing and type of window (single, double, laminated, or triple glazing; or double window), *b*) bedroom orientation (facing the postal address street/side street/backyard), and *c*) frequency of closing windows during sleeping hours (always/often/seldom/never). Availability of shutters and use of ear plugs was rarely reported and not used in this study.

We combined outdoor traffic *L*_night_ with the questionnaire data to calculate two estimates of “personal” noise exposure:

Outdoor traffic *L*_night_ at bedroom façade (step *a*). On the basis of refined modeling techniques for shielded areas (Salomons et al. 2009), we subtracted 20 dB(A) from the outdoor noise estimates at the postal address to obtain noise levels at the bedroom façade where participants slept. We left outdoor estimates unchanged for bedrooms facing the postal address street or a side street. Noise levels at the side street façade were difficult to quantify, and we assumed they were similar to those at the postal address street.

Indoor traffic *L*_night_ at the bedroom (step *b*). We corrected the outdoor traffic *L*_night_ levels at the bedroom façade (step *a*, above) by subtracting an insulation factor that we calculated according to the reported window types and the frequency of keeping windows closed at night. This is described in the *Good Practice Guide on Noise Exposure and Potential Health Effects* (European Environment Agency 2010). Levels of window insulation are commonly derived from laboratory acoustical measurements, and standard values are described in the Spanish Building Code and complementary technical information (Spanish Government 2010; Tremco Ltd. 2004). The insulation factors when “Always closing windows” (100% time) were –30 dB(A) for single and double glazing and –40 dB(A) for sound-proofed windows (triple or laminated glazing or double windows). If windows were “often” (75% of the time), “seldom” (25%), and “never” closed, the resulting insulation factors were –21 dB(A), –16 dB(A), and –15 dB(A), respectively, with no further contribution of the specific insulation of each window type.

We followed step *b* to obtain indoor railway *L*_night_ from outdoor estimates.

We also derived individual outdoor levels of annual average nitrogen dioxide (NO_2_) concentrations (micrograms per cubic meter) at each geocoded address with a land use regression model (LUR) derived in 2010 for Girona, as described elsewhere (Rivera et al. 2013). Briefly, the LUR was based on a dense network of residential outdoor NO_2_ measurements (years 2007–2009). The main predictor variables were the height above street and traffic-related variables within different buffers (from 25- to 1,000-m radii) around the sampling locations. The coefficient of determination (*R*^2^) of the model was 0.63.

*Other data collection*. Based on questionnaires we also assessed smoking (smoker/ex-smoker of > 1 year/never smoker), weekly leisure time physical activity (in metabolic equivalents) with Minnesota’s questionnaire (Elosua et al. 2000), daily alcohol intake (grams per day), adherence score to Mediterranean diet (lowest to highest, from 10 to 30) (Schröder et al. 2004), family history of cardiovascular disease (yes/no), living alone (yes/no), and hearing loss (no/mild/severe). We assessed socioeconomic status at the individual level with educational level (university/secondary/primary/illiterate) and occupation (employed/homemaker-inactive/retired/unemployed), and at the census tract of residences with the deprivation index (Domínguez-Berjón and Borrell 2005). We defined diabetes as fasting blood glucose levels ≥ 126 mg/dL or reported treatment with antidiabetic drugs; body mass index (BMI) as weight/height squared (kilograms per meter squared); intake of anxiolytics as having ever taken tranquilizers, sedatives, anxiety pills, sleeping pills, or muscle relaxants in the last two weeks (yes/no); and CVD as having ever had a cardiovascular event (myocardial infarction or stroke) or cardiovascular-related surgery intervention (yes/no).

We derived daily means of NO_2_ (micrograms per cubic meter) and temperature (degrees Celsius) 0–3 days before the day of examination (lags 0–3) at an urban background station from the regional air quality and meteorology monitoring networks to control for the short-term effects of temperature and air pollution on BP (Servei de Vigilància i Control de l’aire 2008; Servei Meteorològic de Catalunya 2011). Season was categorized as winter (January–March), spring (April–June), summer (July–September), and autumn (October–December).

*Statistical analysis*. We performed descriptive analyses of all variables, assessed their linearity against the outcomes with generalized additive models, and transformed them accordingly. We excluded missing observations on the outcomes, exposure, and covariates of the main models (*n* = 141, 6.8%), resulting in 1,926 cases with characteristics similar to those of the original sample. The inclusion of confounders in the multivariate logistic regression (for hypertension) and linear regression models (for BP) was based on the hypothesized causal pathway of traffic noise and air pollution on hypertension (Fuks et al. 2011) and previous literature. All single and multi-exposure models were controlled for age, age squared, sex, educational level, physical activity, diet, alcohol consumption, smoking, diabetes, BMI, deprivation, railway noise, and short-term effects of daily temperature (lag 0) on measured BP. Occupational status, living alone, temperature at lags 1–3, instead of lag 0, and daily NO_2_ (lags 0–3) did not contribute further to models (i.e., effect estimates changed < 10%). We additionally adjusted for BP-lowering treatment in models for BP and checked regression diagnostics. Effect estimates changed < 10% by further inclusion of potential intermediates (traffic noise annoyance, family history of cardiovascular death, heart rate, and CVD), so these were not considered (data not shown).

We also assessed linear threshold models assuming noise effects to start at 30 dB(A) indoors, the recommended indoor noise levels at night (WHO 2009). For this, we created a new variable by subtracting 30 dB(A) to the noise levels and giving the value zero to the resulting negative values. This new variable was then used as the exposure variable in the models.

We tested population characteristics that could modify the association between traffic noise (indoors) and hypertension by including an interaction term (i.e., evaluated categorical or continuous variable × indoor traffic noise) in multivariate models and checking its statistical significance (i.e., *p*-value of interaction term) as well as the stratum-specific effect estimate of the studied association. The evaluated ordinal variables were coded with consecutive numbers, multiplied by indoor traffic noise, and the resulting continuous variable was used in the models to test for trends. We evaluated age, sex, educational level, BMI, diabetes, traffic annoyance, noise sensitivity with a cut-off at the median, hearing loss, and intake of anxiolytic medication. Anxiolytics have been linked to transportation noise exposure (Floud et al. 2011), and their mechanism of action may directly affect the suggested stress pathway by which noise affects CVD.

Because of the rather high correlation between outdoor traffic noise and NO_2_, we evaluated collinearity in two-exposure models with the variance inflation factor (VIF). A simulation study to assess the effects of collinearity on effect estimates was implemented by repeatedly (10,000 times) simulating data sets and fitting our final model. All final model predictors were simulated from a multivariate normal distribution with mean and covariance matrices as observed in the original data set; SBP was simulated using the regression equation obtained in our study plus normally distributed random error with mean zero and variance equal to the estimated residual variance in the original data set. The correlation between estimated coefficients for outdoor (or indoor) traffic *L*_night_ and NO_2_ were calculated. We carried out the same procedure with indoor traffic *L*_night_.

We reported estimated changes in the outcomes per 5 dB(A) for all noise indicators and per 10 μg/m^3^ for NO_2_, unless otherwise specified. We defined statistical significance at an alpha level of 0.05.

Analyses were performed with Stata 12.0 (StataCorp, College Station, TX, USA) and R version 2.12 (http://www.r-project.org/).

## Results

The main characteristics of the study sample are summarized in Table 1 and in the Supplemental Material, Table S1. The prevalence of hypertension was 36.6%, and 24.1% of the sample took BP-lowering medication. The median age of the participants was 56 years, and 45.5% were male. As expected, compared with nonhypertensive participants, hypertensive participants were older (median, 63 vs. 52 years old, respectively) and had a higher prevalence of diabetes and hearing loss. There were also fewer current smokers among hypertensive participants. Hypertensive participants were exposed to slightly higher levels of NO_2_ [median, interquartile range (IQR): 26.3 (11.2) vs. 27.4 (12.2) μg/m^3^] and noise. The median levels of outdoor traffic *L*_night_ and *L*_night_ at the bedroom façade were almost 30 dB(A) higher than indoors [56.7, 53.5, and 27.1 dB(A), respectively], but outdoor *L*_night_ had a narrower IQR than the other two.

**Table 1:** Main characteristics of the study sample.

Characteristic	Total (*n* = 1,926)	Nonhypertensive (*n* = 1,222)	Hypertensive (*n* = 704)	*p*-Value^*a*^
Continuous variables [median (IQR)]
Systolic blood pressure (mmHg)	123.0 (24.0)	117.0 (15.0)	143.0 (21.0)	< 0.001
Diastolic blood pressure (mmHg)	78.0 (13.0)	75.0 (10.0)	86.0 (13.0)	< 0.001
Age (years)	56.0 (18.0)	52.0 (15.0)	63.0 (15.0)	< 0.001
Mediterranean diet adherence score^*b*^	20.0 (4.00)	20.0 (4.00)	20.0 (4.00)	0.483
Deprivation index^*c*^	–1.95 (0.91)	–2.01 (1.00)	–1.82 (1.30)	< 0.001
Outdoor annual average NO_2_ (μg/m^3^)	26.8 (11.5)	26.3 (11.2)	27.4 (12.2)	0.017
Outdoor traffic *L*_night_ [dB(A)]	56.7 (6.80)	56.5 (6.70)	57.4 (7.00)	< 0.001
Outdoor traffic *L*_night_ at bedroom façade [dB(A)]	53.5 (17.2)	53.4 (16.9)	53.7 (17.6)	0.03
Indoor traffic *L*_night_ at bedroom [dB(A)]	27.1 (16.2)	26.9 (15.8)	27.6 (17.2)	0.061
Indoor railway *L*_night_ at bedroom [dB(A)]	10.5 (21.6)	10.0 (21.4)	11.1 (22.0)	0.572
Noise sensitivity score (10–60)^*d*^	33.0 (17.0)	34.0 (17.0)	30.0 (17.0)	< 0.001
Categorical variables [*n* (%)]
Male sex	876 (45.5)	493 (40.3)	383 (54.4)	< 0.001
BMI (kg/m^2^)
< 20	68 (3.50)	60 (4.90)	8 (1.10)	< 0.001
20–25	605 (31.4)	477 (39.0)	128 (18.2)
25.1–30	851 (44.2)	517 (42.3)	334 (47.4)
> 30	402 (20.9)	168 (13.7)	234 (33.2)
Educational level
University or similar	596 (30.9)	438 (35.8)	158 (22.4)	< 0.001
Secondary	618 (32.1)	428 (35.0)	190 (27.0)
Primary	681 (35.4)	346 (28.3)	335 (47.6)
Illiterate	31 (1.60)	10 (0.80)	21 (3.00)
Smoking
Never smokers	981 (50.9)	613 (50.2)	368 (52.3)	0.004
Smokers	406 (21.1)	285 (23.3)	121 (17.2)
Former smokers	539 (28.0)	324 (26.5)	215 (30.5)
Diabetes, yes	261 (13.6)	97 (7.90)	164 (23.3)	< 0.001
Bedroom orientation, back yard^*e*^	582 (30.2)	369 (30.2)	213 (30.3)	0.978
Closing windows, yes^*f*^	885 (46.0)	574 (47.0)	311 (44.2)	0.236
Protections, yes^*g*^	666 (34.6)	419 (34.3)	247 (35.1)	0.723
Traffic annoyance (points)
None (0)^*h*^	1,198 (62.6)	737 (60.6)	461 (66.0)	0.065
Moderate (1–5)	549 (28.7)	368 (30.3)	181 (25.9)
High (6–10)	168 (8.80)	111 (9.10)	57 (8.20)
Anxiolytics, yes	425 (22.2)	239 (19.6)	186 (26.6)	< 0.001
^***a***^Chi-square test and Kruskal–Wallis test for strata of hypertension with categorical or continuous variables, respectively. ^***b***^10 (lowest) and 30 (highest) adherence to diet. ^***c***^High deprivation corresponds to high values. ^***d***^Higher noise sensitivity with higher values; 10.8% missing observations. ^***e***^Versus bedroom facing postal address street or side-street. ^***f***^Yes: always close windows (vs. no: never, seldom, often close windows). ^***g***^Sound-proofed windows or bedroom facing the backyard. ^***h***^*n* < 1,926 (< 1% missing observations).				

Outdoor NO_2_ concentrations were highly correlated with outdoor levels of traffic *L*_night_ (Spearman’s correlation coefficient, *r* = 0.75), but not with traffic *L*_night_ at the bedroom façade and indoor traffic *L*_night_ (0.39 and 0.23, respectively) (Table 2).

**Table 2:** Spearman correlations*^a^* between annual average home outdoor NO_2_ levels and outdoor and indoor traffic noise levels (*L*_night_) in the city of Girona (*n* = 1,926).

Variable	Outdoor NO_2_	Outdoor *L*_night_	Outdoor *L*_night_ at façade	Indoor *L*_night_
Outdoor annual average NO_2_ (μg/m^3^)	1.00			
Outdoor *L*_night_ [dB(A)]	0.75	1.00		
Outdoor *L*_night_ at bedroom façade [dB(A)]	0.39	0.55	1.00	
Indoor *L*_night_ [dB(A)]	0.23	0.35	0.78	1.00
^***a***^All correlations are statistically significant at α < 0.001.				

Participants who always closed windows and had noise protections (i.e., with bedroom facing the backyard or sound-proofed windows; 15% of the participants) were exposed to slightly higher median outdoor *L*_night_ levels [57.2 dB(A)] compared with those who had none of these noise-reducing measures [56.1 dB(A), 34%], or those who only closed windows [56.9 dB(A), 31%] or only had protections [56.9 dB(A), 20%] (Kruskal–Wallis test *p*-value = 0.044). Median outdoor traffic *L*_night_ levels were also higher for those reporting higher traffic annoyance [not annoyed: 56.1 dB(A); moderately: 57.3 dB(A); highly annoyed: 58.1 dB(A); Kruskal–Wallis test *p*-value < 0.001], but not for those with higher noise sensitivity.

*Traffic* L*_night_, NO_2_, and high BP*. In single-exposure models, outdoor traffic *L*_night_ and NO_2_ were associated with prevalent hypertension [OR = 1.18; 95% CI: 1.05, 1.32 per 5 dB(A) and OR = 1.16; 95% CI: 0.99, 1.36 per 10 μg/m^3^, respectively] (Table 3). When combining both factors in two-exposure models, the association for outdoor traffic *L*_night_ was similar, whereas that for NO_2_ was attenuated (OR = 0.98; 95% CI: 0.79, 1.22). In contrast, we observed associations of NO_2_, traffic *L*_night_ at the bedroom façade and indoor traffic *L*_night_ with hypertension that were not confounded by noise or NO_2_, correspondingly. Relationships with indoor traffic *L*_night_ and NO_2_ did not reach statistical significance (OR = 1.06; 95% CI: 0.99, 1.13; *p* = 0.073) and (OR = 1.16; 95% CI: 0.99, 1.36; *p* = 0.058), respectively.

**Table 3:** Estimated change in the prevalence of hypertension, SBP, and DBP (mmHg) per increasing*^a^* residential levels of traffic noise (*L*_night_) and annual average outdoor NO_2_ (*n* = 1,926).

Models^*b*^	Hypertension [OR (95% CI)]		SBP [β (95% CI)]		DBP [β (95% CI)]	
	*L*_night_	NO_2_	*L*_night_	NO_2_	*L*_night_	NO_2_
Outdoor model^*c*^
Single-exposure	1.18 (1.05, 1.32)**	1.16 (0.99, 1.36)*	0.51 (–0.24, 1.25)	1.19 (0.17, 2.21)**	0.20 (–0.23, 0.63)	0.55 (–0.04, 1.14)*
Multi-exposure	1.19 (1.02, 1.40)**	0.98 (0.79, 1.22)	–0.20 (–1.25, 0.84)	1.39 (–0.05, 2.82)*	–0.17 (–0.77, 0.44)	0.71 (–0.12, 1.54)*
Façade model^*d*^
Single-exposure	1.08 (1.01, 1.15)**	1.16 (0.99, 1.36)*	0.42 (0.00, 0.83)**	1.19 (0.17, 2.21)**	0.08 (–0.16, 0.32)	0.55 (–0.04, 1.14)*
Multi-exposure	1.07 (1.01, 1.14)**	1.14 (0.97, 1.33)	0.36 (–0.06, 0.77)*	1.07 (0.04, 2.10)**	0.06 (–0.18, 0.29)	0.53 (–0.06, 1.13)*
Indoor model^*e*^
Single-exposure	1.06 (0.99, 1.13)*	1.16 (0.99, 1.36)*	0.71 (0.28, 1.14)**	1.19 (0.17, 2.21)**	0.09 (–0.16, 0.34)	0.55 (–0.04, 1.14)*
Multi-exposure	1.06 (0.99, 1.13)*	1.16 (0.99, 1.36)*	0.72 (0.29, 1.15)**	1.23 (0.21, 2.25)**	0.10 (–0.15, 0.34)	0.56 (–0.03, 1.14)*
Single-exposure models were for NO_2_ or the corresponding traffic noise indicator. Multi-exposure models were adjusted for NO_2_ and the corresponding traffic noise indicator. ^***a***^Per 5 dB(A) of traffic *L*_night_ and 10 μg/m^3^ of NO_2_. ^***b***^All models were adjusted for age, age squared, sex, education, Mediterranean diet, exercise, alcohol consumption, smoking, BMI, diabetes, deprivation, daily temperature, and indoor railway noise. BP models were additionally adjusted for BP-lowering treatment. ^***c***^Noise indicator: outdoor traffic *L*_night_. ^***d***^Noise indicator: outdoor traffic *L*_night_ at the bedroom façade. ^***e***^Noise indicator: indoor traffic *L*_night_ at the bedroom. *p < 0.1. **p < 0.05.						

Regarding SBP, we observed a nonsignificant increment of 0.51 mmHg (95% CI: –0.24, 1.25) per 5-dB(A) increase of outdoor traffic *L*_night_, and a significant increment of 1.19 mmHg (95% CI: 0.17, 2.21) per 10 μg/m^3^ of NO_2_ in single-exposure models (Table 3). In contrast, in two-exposure models, the point estimate for noise was negative (β = –0.20; 95% CI: –1.25, 0.84), whereas the relationship with NO_2_ became stronger but less precise (β = 1.39; 95% CI: –0.05, 2.82). This confounding was not present in two-exposure models for indoor traffic *L*_night_ and NO_2_, and both yielded statistically significant associations with SBP, respectively: β = 0.72 (95% CI: 0.29, 1.15) and β = 1.23 (95% CI: 0.21, 2.25). *L*_night_ at the bedroom façade was positively associated with SBP, although the association did not reach statistical significance after adjustment for NO_2_ (β = 0.36; 95% CI: –0.06, 0.77). Finally, we observed an association between NO_2_ and DBP, but not with traffic *L*_night_.

*Threshold effect for indoor traffic noise*. After we applied a threshold at 30 dB(A), indoor traffic *L*_night_ yielded stronger associations with hypertension: OR = 1.14 (95% CI: 0.99, 1.31) and particularly with SBP: β = 1.27 (95% CI: 0.34, 2.20) (tested in two-exposure models). Indeed, we observed a slight departure from linearity with a potential threshold at 30 dB(A) with SBP (see Supplemental Material, Figure S2). The associations between NO_2_ and the outcomes in these models remained similar (see Supplemental Material, Table S2).

**Figure 1:**
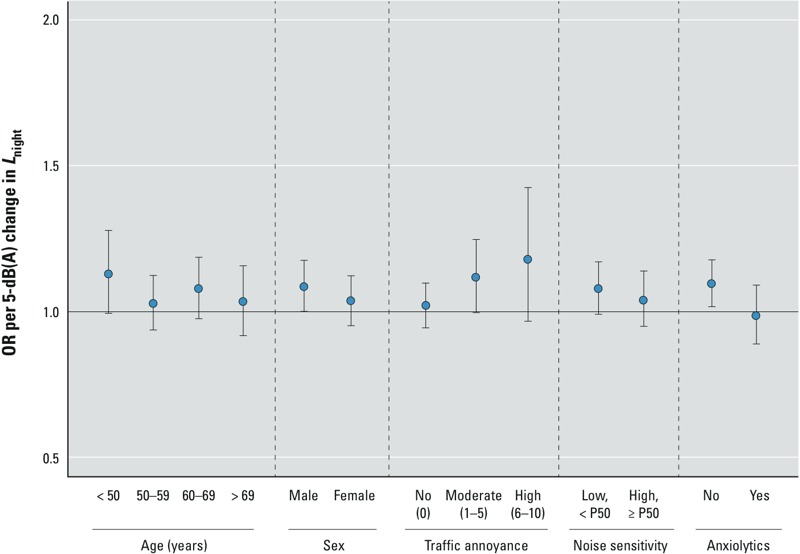
Estimated change in prevalent hypertension per increment of 5 dB(A) in annual average levels of nighttime indoor traffic noise at the bedroom by subgroups of population characteristics (*n* = 1,926). P50, 50th percentile. Each multivariate logistic regression model was adjusted for the corresponding interaction term, one at a time, and annual average NO_2_ levels, age, age squared, sex, education, Mediterranean diet, exercise, alcohol consumption, smoking, BMI, diabetes, deprivation, daily temperature, and indoor railway *L*_night_.

*Effect modifiers*. Associations between indoor traffic noise and hypertension were seen in subjects not taking anxiolytics (OR = 1.10; 95% CI: 1.02, 1.18) and not in those taking anxiolytics (OR = 0.99; 95% CI: 0.89, 1.09; *p*-value of interaction = 0.054). There was also a trend toward stronger associations between indoor traffic *L*_night_ and hypertension with increasing reported traffic annoyance: no annoyance (OR = 1.02; 95% CI: 0.95, 1.10), moderate annoyance (OR = 1.12; 95% CI: 1.0, 1.25), and high annoyance (OR = 1.18; 95% CI: 0.97, 1.43); all *p*-values of interaction (categorical variable) = 0.141, all *p*-values of interaction (continuous variable) = 0.033, all *p*-values for trend = 0.052. We found no indication for interactions by age, sex, educational level, BMI, diabetes, noise sensitivity, and hearing loss (all *p*-values of interaction > 0.31). See Figure 1 and Supplemental Material, Table S3.

*Collinearity between traffic* L*_night_ and NO_2_*_._ The VIFs for outdoor traffic *L*_night_ and NO_2_ when combined in two-exposure models were < 2.8 (common rule of thumb for collinearity is VIF > 5 or > 10).

The average beta coefficients of the 10,000 simulations were for NO_2_, β = 1.19 and for outdoor traffic *L*_night_, β = 0.51, and their Pearson correlation was –0.70 (see Supplemental Material, Figure S1A). In contrast, the resulting correlation between the simulated beta coefficients of NO_2_ and indoor traffic *L*_night_ was 0.03 (see Supplemental Material, Figure S1B).

## Discussion

This study combined long-term estimates of outdoor traffic noise levels at night (*L*_night_) with information on bedroom orientation and measures to abate noise to derive an estimate of indoor traffic noise levels at each participant’s bedroom. Besides attempting to get a more accurate estimate of the true relevant exposure, accounting for noise-reducing factors decreases the correlation observed between outdoor traffic noise and NO_2_ levels (a marker of traffic-related air pollution). Thus it helps to disentangle the associations of these traffic-related stressors with high BP. Few studies to date have considered this mutual confounding on high BP (Coogan et al. 2012; de Kluizenaar et al. 2007; Fuks et al. 2011; Sørensen et al. 2011, 2012), and none have used indoor noise estimates. Moreover, few studies analyzed both hypertension and BP. We observed associations between indoor traffic noise and both hypertension and SBP, and between NO_2_ and hypertension, SBP, and DBP. The associations of indoor traffic noise were not confounded by NO_2_, and vice versa. In contrast, results for outdoor traffic *L*_night_ were less consistent, and associations between outdoor traffic *L*_night_ and NO_2_ with the outcomes showed opposite tendencies after mutual adjustment.

The less consistent findings for outdoor traffic *L*_night_ agreed with the literature, which indicates associations with hypertension, but limited evidence with BP (Babisch 2006; Dratva et al. 2012; Sørensen et al. 2011; van Kempen and Babisch 2012). Regarding the estimated effect size, a recent meta-analysis reported an OR of 1.03 (95% CI: 1.01, 1.06) per 5-dB(A) change of daytime traffic noise (van Kempen and Babisch 2012). We observed a higher OR of 1.19 (95% CI: 1.02, 1.40), which may reflect a residual confounding by traffic-related air pollution in our study area, due to the high correlation between the two outdoor factors, and thus, the inability to disentangle associations even after adjustment for NO_2_, as discussed below.

In contrast, indoor traffic *L*_night_ was suggestively associated with hypertension (OR = 1.06; 95% CI: 0.99, 1.13, *p*-value = 0.073) and the estimated effect size was closer to the above-mentioned meta-analysis (van Kempen and Babisch 2012). Furthermore, it was also associated with SBP. The null association for DBP was previously observed by Sørensen et al. (2011) with outdoor traffic noise. Further research is needed to clarify whether the chronic noise–stress biological pathway may promote vascular changes resulting in isolated increased SBP (Black and Elliott 2013).

To our knowledge, only one study has estimated indoor traffic noise (as a categorical variable) according to two terms: room orientation and always closing windows. Only the indoor estimates yielded an increase in the risk of ischemic heart disease, though it was not statistically significant (Babisch et al. 1999). Our assessment further computed the frequency of opening windows, and used more precise, continuous noise estimates with a wider exposure contrast. The other few attempts to account for noise-reducing factors consisted of stratification or interaction analysis by these factors on the noise–hypertension relationship, and only one study addressed this issue comprehensively (Babisch et al. 2012). However, results have been heterogeneous. We assessed similar interaction analyses with closing windows, protections, and a combination of the two, and did not identify differences among groups (data not shown). Stratified analyses have lower statistical power and might result in bias and spurious findings due to multiple comparisons. Furthermore, people may combine noise-reducing remedies, and findings for specific measures might be difficult to interpret if they co-vary with other shielding elements, annoyance, or outdoor traffic noise and air pollution levels.

Our findings for long-term exposure to near-road pollution (NO_2_) also agreed with the emerging literature, which indicates associations with BP (Chuang et al. 2011; Dong et al. 2013; Foraster et al. 2014; Fuks et al. 2011; Schwartz et al. 2012), although not in all studies (Sørensen et al. 2012). Furthermore, we also observed a borderline statistically significant association for hypertension, which was independent of indoor traffic noise, but tended to the null when adjusting for outdoor traffic *L*_night_. To our knowledge, the association between NO_2_ and prevalence of hypertension was observed in only two studies (Dong et al. 2013; Johnson and Parker 2009), whereas other studies found null or inverse effects (Foraster et al. 2014; Fuks et al. 2011; Sørensen et al. 2012). The evidence is more consistent for incidence of hypertension, but only based on two studies (Coogan et al. 2012; Sørensen et al. 2012).

In this study, the beta coefficients of outdoor traffic *L*_night_ and NO_2_ tended to show opposite associations when combined in two-exposure models. We observed a Spearman correlation coefficient of 0.75 between outdoor traffic *L*_night_ and NO_2_. However, according to the VIF and the commonly used thresholds, the tendencies were not explained by collinearity.

To further understand this issue, we implemented a simulation. The simulation showed unbiased average regression coefficients for the association of NO_2_ and outdoor traffic *L*_night_ with SBP after 10,000 replications. This indicates that results from multiple studies (i.e., a meta-analysis) using linear regression and even with an NO_2_–outdoor *L*_night_ correlation of 0.75 would provide unbiased estimates on average. However, the correlation between regression coefficients was –0.70. In other words, those individual replicates of the study finding a high regression coefficient for NO_2_ found a low coefficient for outdoor traffic *L*_night_, and vice versa (see Supplemental Material, Figure S1A). In particular, around 15.1% of studies had a reversed sign for outdoor traffic noise. In the current study, the tendency could be strong enough to reverse the sign of one of the two exposures. Similar results are expected in other studies of similar size and correlation (around 0.7 or higher) between NO_2_ and noise. Therefore there is a risk that literature reporting an association for NO_2_ does not find an association for outdoor traffic noise and vice versa, making it difficult to disentangle associations. This might have happened in three of the few studies combining both stressors, which observed a slight negative confounding, including a recent study of our group that focused on NO_2_ and adjusted for outdoor traffic *L*_night_ as the only available exposure marker (de Kluizenaar et al. 2007; Foraster et al. 2014; Sørensen et al. 2012).

The present study further showed that these opposite tendencies in beta coefficients disappeared when assessing markers of personal exposure at the bedroom façade, which were less correlated with NO_2_ (Table 3). This was also confirmed in the simulation study by a null correlation between the beta coefficients of indoor traffic *L*_night_ and NO_2_ (see Supplemental Material, Figure S1B). This underscores the need for appropriate exposure measurements for both noise and air pollution to adequately disentangle their associations with common end points—to avoid spurious correlations and thus spurious adjustment patterns when one factor (noise in our case) is a poor proxy of exposure.

*Threshold effects for indoor traffic* L*_night_*_._ The association of indoor traffic noise with hypertension and SBP was stronger when we assumed a 30-dB(A) threshold effect for indoor traffic noise. Although departures from linearity were observed only for SBP (see Supplemental Material, Figure S2), a threshold might be possible because indoor noise sources at nighttime could well reach 30 dB(A), thus partly or totally masking the contribution of traffic noise levels < 30 dB(A) indoors. This low threshold indicates that even low traffic noise levels may affect BP and agrees with the WHO recommendations for nighttime noise at bedrooms [30 dB(A)] (WHO 2009).

*Effect modification*. We observed no association between indoor traffic *L*_night_ and hypertension among participants taking anxiolytics, which might indicate that anxiolytics block the stress response by which noise affects BP. This agrees with a laboratory study reporting fewer noise-induced sleep responses with intake of anxiolytic medication (Cluydts et al. 1995).

We also observed that increasing noise annoyance may potentially lead to stronger associations between indoor traffic noise and hypertension (Figure 1). Few studies to date have analyzed this pattern, which could relate to an interaction between the proposed direct and indirect mechanistic pathways of noise (Babisch et al. 2013).

Finally, we could not confirm previous reports of stronger associations in some age groups or in men (van Kempen and Babisch 2012).

*Strengths and limitations*. In this study, we derived markers of traffic noise exposure at the bedroom façade and indoors at night from questionnaire data on noise-reducing factors and the best available literature on insulation (European Environment Agency 2010; Salomons et al. 2009; Spanish Government 2010; Tremco Ltd. 2004). We acknowledge that these corrections may have introduced some error, resulting in less precise or biased estimates, which are difficult to predict. For instance, although we deducted standard values to adjust for window type, the true insulation provided by the different windows may vary because it also depends on proper window seals. Nevertheless, a small proportion of the participants had sound-proofed windows (4.5%), and still 54% opened windows to some degree (a factor we also considered), thus heavily reducing the effect of window insulation. Besides, home construction is quite homogeneous in Girona, thus possibly yielding similar insulations in backyards. However, models that estimate noise at all building façades are needed to improve precision. In summary, in this study, both markers of noise exposure at the bedroom (particularly the indoor marker) provided more plausible results than outdoor noise at the postal address. Even though our novel questionnaire-based assessment seems suitable, future studies should confirm our results and could even improve questionnaires to obtain even more precise information.

We emphasize that the exposure misclassification now addressed for noise does not necessarily apply to the same extent to air pollution. Many exposure studies confirmed that indoor concentrations of pollutants from outdoor origin, as well as traffic-related particulate matter components such as black smoke (Gotschi et al. 2002), are highly correlated with the outdoor concentrations (Chen and Zhao 2011). This may particularly apply to Girona, where only 46% of participants always closed windows at night and where ventilation during the day is expected given the mild temperatures.

We relied on a detailed noise and LUR model for Girona. However, our exposure models were derived for a specific year and the current residence only, which could lead to exposure misclassification. Nevertheless, the city had no major changes in traffic during the years before the exposure assessment; therefore, we expect spatial distributions of both environmental factors to represent long-term exposure. Moreover, residential mobility was low, and restricting the analyses to nonmovers up to 10 years before the baseline examination had no influence on results (data not shown).

Regarding the noise questionnaire, responses referred to the time of the follow-up visit, but participants were nonmovers. Thus, we expect that most responses represent exposure at baseline. However, because noise-reducing factors may come later as a consequence of annoyance or disease, we may have underestimated the baseline exposure and the true associations for some participants. Finally, although reported noise-reducing remedies could vary across seasons, season of reexamination did not influence the association between indoor traffic noise and the outcomes (*p*-values of interaction > 0.34).

We assessed nighttime, a particularly susceptible period for the adverse health effects of noise (WHO 2009). Daytime indoor traffic noise should be estimated in rooms where activities may be disturbed, and this may be more difficult to determine. Moreover, we expect the daytime to account for a smaller proportion of the total relevant exposure. Besides, although long-term average traffic noise levels (available from current models) could be representative of peak values, given their high correlation (WHO 2009), peaks might be more disturbing, and future efforts are needed to characterize and assess their health impact.

We also considered a comprehensive set of adjustment variables which had little influence on coefficients. However, residual confounding could always remain, particularly from other traffic-related air pollutants not well captured with our marker (NO_2_).

As previously argued (Foraster et al. 2014), no perfect method exists to deal with the intake of BP-lowering medication in BP analyses. The stratified analyses by medication did not indicate a strong masking of the studied associations by medication in the treated group (see Supplemental Material, Table S4). Actually, these associations were even stronger in this group, suggesting that the most affected individuals tended to be medicated. Moreover, our results for BP were robust across all alternative methods (Tobin et al. 2005) in the entire sample, which is reassuring. Thus, for simplicity and to increase statistical power in this rather small study, we retained all study participants and presented the results with the commonly used approach of adjustment for medication.

A main limitation of this study was its cross-sectional design, so distinguishing causes from effects is not possible. Nevertheless, results for indoor traffic noise and NO_2_ seem plausible and in line with the biological mechanisms (Babisch 2011; Brook et al. 2009). In addition, given the rather small sample size, we may lack statistical power, particularly for the binary variable of hypertension and the stratified analyses.

Another limitation is that we assessed BP with standard protocols of repeated measurements during one single examination, which does not allow a clinical diagnosis of hypertension. Nevertheless, we know that at least 50% of those with high BP in our cohort confirmed their hypertension in the following years (Foguet et al. 2008). Furthermore, most hypertensive subjects were classified according to their antihypertensive treatment, and we selected the last BP measurement available to minimize the “white-coat” effect. Despite the efforts to minimize variability in BP, we cannot exclude a remaining nondifferential misclassification, which would bias results toward the null.

Finally, we selected participants attending the follow-up, so some self-selection of healthier participants might have occurred, potentially biasing results toward the null too.

*Public health implications*. Even low levels of both traffic-related factors (noise and air pollution) may contribute to hypertension, and thus to CVD—a primary cause of morbidity and mortality. Although estimated effect sizes were small, these stressors are ubiquitous, so decreasing their levels could benefit millions of people. Our results further suggest that individual measures against noise in Girona were insufficient: Whether current noise protections reduce BP is unclear (Babisch et al. 2012).

## Conclusions

In this cross-sectional study we identified an association between long-term exposure to indoor traffic noise at night and both prevalent hypertension and SBP, as well as an association between long-term exposure to NO_2_—a marker of traffic-related air pollution—and both prevalent hypertension and BP. These results should be further confirmed, but they underscore the relevance of using detailed exposure assessment to identify the independent associations of traffic noise and traffic-related air pollution (Künzli 2013) on common outcomes. Questionnaires on measures against noise could be a useful tool to derive indoor noise markers in future studies.

## Supplemental Material

(313 KB) PDF
