# Supplementary material for: High Blood Pressure and Long-Term Exposure to Indoor Noise and Air Pollution from Road Traffic
Source: Environ Health Perspect. 2014 Jul 8;122(11):1193–200. doi: 10.1289/ehp.1307156 (PMC4216159; doi:10.1289/ehp.1307156)
Supplement: (313 KB) PDF [file ehp.1307156.s001.508.pdf]

Supplemental Material

High Blood Pressure and Long-Term Exposure to Indoor Noise and Air Pollution from Road Traffic

Maria Foraster, Nino Künzli, Inmaculada Aguilera, Marcela Rivera, David Agis, Joan Vila, Laura Bouso, Alexandre Deltell, Jaume Marrugat, Rafel Ramos, Jordi Sunyer, Roberto Elosua, and Xavier Basagaña

**Table of Contents**	**Page**
**Table S1**	**2**
**Table S2**	**3**
**Table S3**	**4**
**Table S4**	**5**
**Figure S1**	**6**
**Figure S2**	**7**

Table S1. Extension of Table 1 on the main characteristics of the study sample (n = 1926).

Characteristic	Total (n=1926)	Non-hypertensive (n=1222)	Hypertensive (n=704)	p-value[a]
**Continuous variables [median (IQR)]**				
Outdoor railway noise levels, L_{night} [dB(A)]	41.8 (15.2)	41.5 (16.0)	42.1 (14.2)	0.558
Daily mean temperature at lag 0 (ºC)	13.8 (12.9)	13.9 (13.1)	13.6 (12.7)	0.589
**Categorical variables [n (%)]**				
Daily alcohol intake (g/l)				
No alcohol	441 (22.9)	282 (23.1)	159 (22.6)	0.033
Little (≤ 20)	1227 (63.7)	796 (65.1)	431 (61.2)	
Moderate (20.1-39.9)	198 (10.3)	114 (9.30)	84 (11.9)	
Excessive (≥ 40)	60 (3.10)	30 (2.50)	30 (4.30)	
Weekly physical activity (MET)				
Tertile 1	630 (32.7)	399 (32.7)	231 (32.8)	0.655
Tertile 2	641 (33.3)	415 (34.0)	226 (32.1)	
Tertile 3	655 (34.0)	408 (33.4)	247 (35.1)	
Hearing loss, no	1188 (65.7)	792 (69.4)	396 (59.5)	0.221
Mild	481 (26.6)	289 (25.3)	192 (28.8)	
Severe	139 (7.70)	61 (5.30)	78 (11.7)	
Living alone, yes	200 (10.4)	119 (9.70)	81 (11.5)	0.221
Season of re-examination, winter	372 (19.3)	230 (18.8)	142 (20.2)	< 0.001
Spring	562 (29.2)	321 (26.3)	241 (34.2)	
Summer	426 (22.1)	305 (25.0)	121 (17.2)	
Autumn	566 (29.4)	366 (30.0)	200 (28.4)	

L_{night}: Long-term average nighttime noise level; MET: Metabolic equivalents.

[a] χ^2 test and Kruskal-Wallis test for strata of hypertension with categorical or continuous variables, respectively.

Table S2. Estimated change in the prevalence of hypertension [odds ratios (95% CI)] and in systolic (SBP) and diastolic (DBP) blood pressure (mmHg) [beta coefficients (95% CI), in mmHg] per 5 dB(A) increase of indoor traffic noise at night (L_{night}) and per 10 μg/m^3 increase of annual average levels of outdoor nitrogen dioxide (NO_2) applying a linear threshold model for indoor noise at 30 dB(A), (n = 1926).

Multi-exposure model	Hypertension	SBP	DBP
Indoor traffic L_{night}	1.14 (0.99, 1.31)*	1.27 (0.34, 2.20)**	0.17 (-0.36, 0.71)
NO_2	1.17 (1.00, 1.37)*	1.25 (0.23, 2.27)**	0.56 (-0.03, 1.15)*

Model adjusted for indoor traffic L_{night}, annual average NO_2, age, age squared, sex, education, Mediterranean diet, exercise, alcohol consumption, smoking, BMI, diabetes, deprivation, daily temperature, BP-lowering medication, and indoor railway L_{night}.

** $p < 0.05$, * $p < 0.1$

Table S3. Estimated change in the prevalence of hypertension per increment of 5dB(A) in annual average levels of indoor traffic noise at night (L_{night}) by subgroups of population characteristics. A subset of the main results in this table is graphically shown in Figure 1.

Interaction term	N	Odds ratio (95% CI)[a]	p-value interaction
Age, < 50	591	1.13 (1.00, 1.28)	0.540[b]
50-59	594	1.03 (0.94, 1.13)	
60-69	421	1.08 (0.98, 1.19)	
> 69	320	1.03 (0.92, 1.16)	
Gender, male	879	1.09 (1.00, 1.18)	0.315
female	1050	1.04 (0.96, 1.12)	
Traffic annoyance, no	1198	1.02 (0.95, 1.10)	0.141[c]
Moderate (1-5)	549	1.12 (1.00, 1.25)	
High (6-10)	168	1.18 (0.97, 1.43)	
Low noise sensitivity, < P50	908	1.08 (1.00, 1.17)	0.498
High noise sensitivity, ≥ P50	810	1.04 (0.96, 1.14)	
Anxiolytics, no	1492	1.10 (1.02, 1.18)	0.054
Yes	425	0.99 (0.89, 1.09)	
Educational level			
University or similar	596	1.06 (0.96, 1.18)	0.454
Secondary	618	1.12 (1.01, 1.25)	
Primary	681	1.03 (0.95, 1.12)	
Illiterate	31	1.18 (0.84, 1.68)	
Diabetes, no	1665	1.07 (0.99, 1.14)	0.798
Yes	261	1.05 (0.93, 1.19)	
Body mass index, < 20	68	0.86 (0.59, 1.25)	0.591
20-25	605	1.04 (0.94, 1.15)	
25.1-30	851	1.08 (1.00, 1.18)	
>30	402	1.05 (0.94, 1.17)	0.559
Hearing loss, no	1188	1.08 (0.99, 1.17)	
Mild	481	1.04 (0.96, 1.15)	
Severe	139	1.16 (0.97, 1.39)	

[a]Each multivariate logistic regression model is adjusted for the corresponding interaction-term, one at a time, and annual average NO_2 levels, age, age squared, sex, education, Mediterranean diet, exercise, alcohol consumption, smoking, BMI, diabetes, deprivation, daily temperature, and indoor railway L_{night} levels. [b]p-value for trend = 0.381 and p-value of interaction for the continuous variable = 0.386. [c]p-value for trend = 0.052 and p-value of interaction for the continuous variable = 0.033.

Table S4. Estimated change in systolic (SBP) and diastolic (DBP) blood pressure [beta coefficients (95% CI), in mmHg] per increments[a] of indoor traffic noise at night (L_{night}) and annual average levels of nitrogen dioxide (NO_2) using different methods to control for BP-lowering medication.

Multi-exposure models[b]	n	SBP, L_{night}	SBP, NO_2	DBP, L_{night}	DBP, NO_2
Non-medicated[c]	1462	0.38 (-0.08, 0.83)	1.10 (0.03, 2.18)**	-0.06 (-0.33, 0.22)	0.59 (-0.06, 1.24)*
Medicated[c]	464	1.64 (0.57, 2.70)**	1.59 (-1.02, 4.20)	0.60 (0.04, 1.15)**	-0.01 (-1.37, 1.35)
Not adjusting for medication[d]	1926	0.75 (0.32, 1.18)**	1.27 (0.23, 2.30)**	0.12 (-0.14, 0.37)	0.58 (-0.02, 1.18)*
Adjusting for medication[e]	1926	0.72 (0.29, 1.15)**	1.23 (0.21, 2.25)**	0.10 (-0.15, 0.34)	0.56 (-0.03, 1.14)*
SBP + 10 mmHg[f]	1926	0.79 (0.33, 1.25)**	1.32 (0.22, 2.42)**	-	-
DBP + 5 mmHg[f]	1926	-	-	0.14 (-0.13, 0.40)	0.60 (-0.03, 1.24)*
Censored regression[g]	1926	0.76 (0.26, 1.26)**	1.24 (0.05, 2.44)**	0.12 (-0.17, 0.42)	0.65 (-0.05, 1.35)*

[a]Per 5 dB(A) of traffic L_{night} and 10 μg/m^3 of NO. [b]All models adjusted for age, age squared, sex, education, Mediterranean diet, exercise, alcohol consumption, smoking, BMI, diabetes, deprivation, daily temperature, indoor railway L_{night}, NO_2, and indoor traffic L_{night}. [c]Medicated means use of BP-lowering medication. [d]Model for the entire study sample, adjusted for variables in [a], but not for BP-lowering medication. [e]Model for the entire study sample, adjusted for variables in [a] and for BP-lowering medication. [f]Addition of 10 mmHg to SBP or 5 mmHg to DBP levels if participant uses BP-lowering medication. [g]Model for the entire study sample. It assumes that had participants under BP-lowering medication not been treated, they would have BP levels.

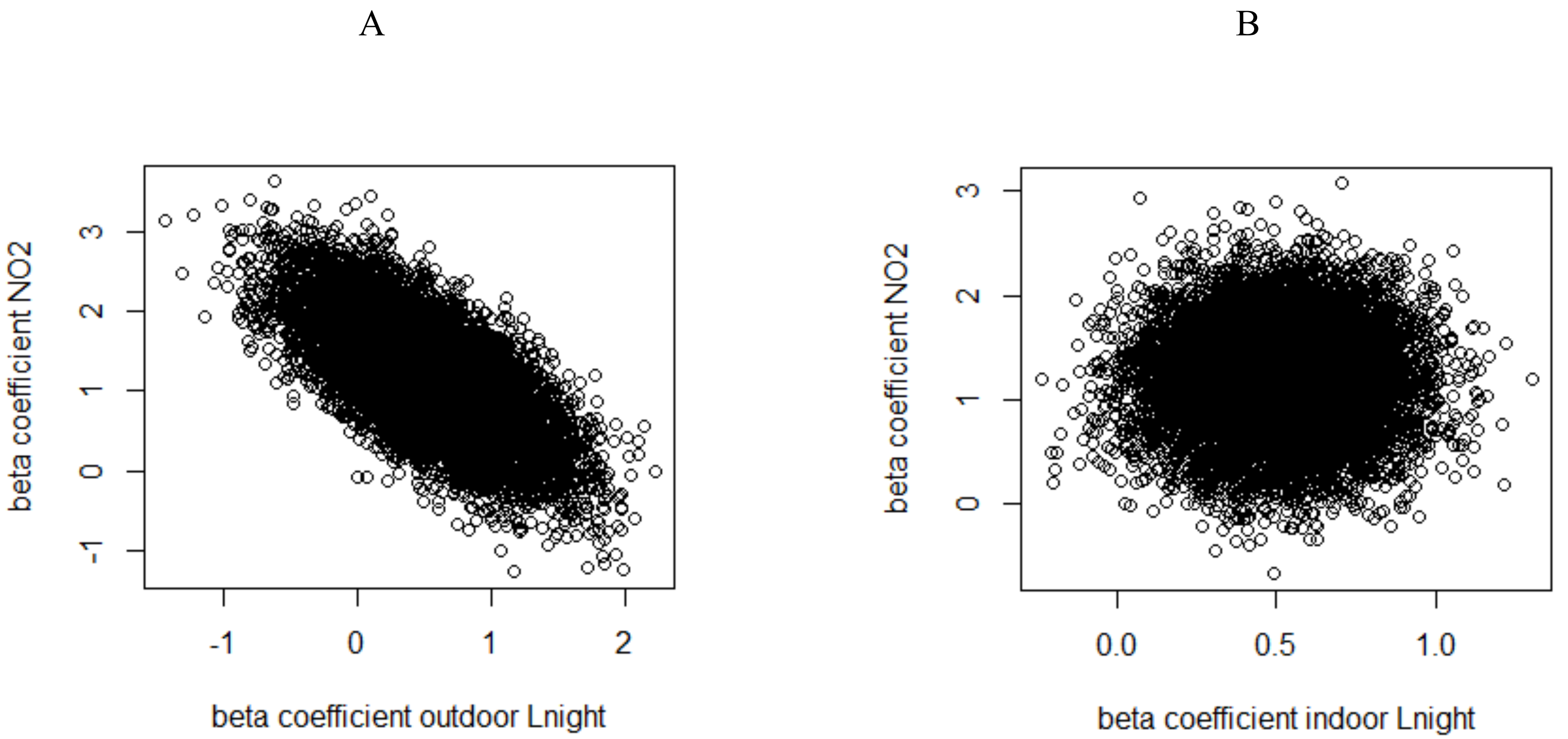

Figure S1. A: Correlation between beta coefficients of NO_2 and *outdoor* traffic L_{night} from 10,000 simulated databases. B: Correlation between beta coefficients of NO_2 and *indoor* traffic L_{night} from 10,000 simulated databases. Multi-exposure regression models for systolic blood pressure and L_{night} and NO_2 adjusted for for: age, age squared, sex, education, Mediterranean diet, exercise, alcohol consumption, smoking, BMI, diabetes, deprivation, daily temperature (lag 0), BP-lowering medication, indoor railway L_{night}.

Hypertension Systolic blood pressure

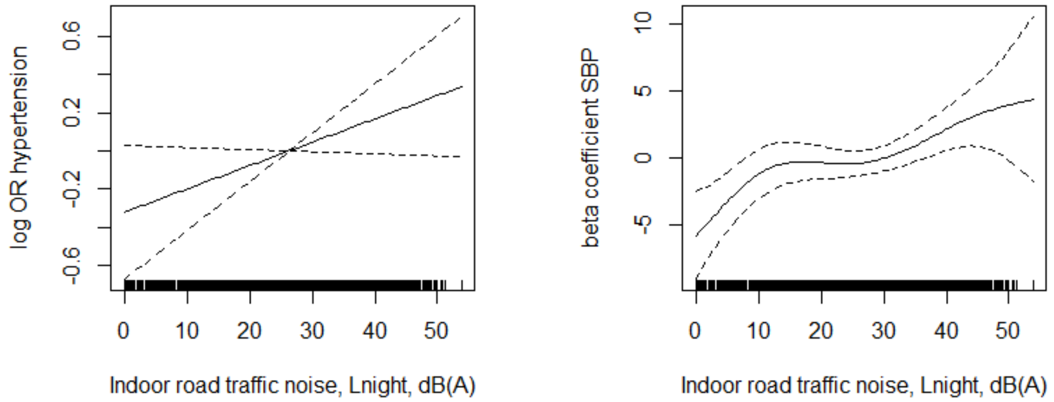

Figure S2. Smooth spline for the effect of 1 dB(A) increase in indoor road traffic L_{night} and 95% confidence intervals in a generalized additive model for hypertension (left) and systolic blood pressure (mmHg) (right). Model adjusted for: age, age squared, sex, education, Mediterranean diet, exercise, alcohol consumption, smoking, BMI, diabetes, deprivation, daily temperature (lag 0), BP-lowering medication, indoor railway L_{night}, and annual average NO_2 (n =1926).
